# Using Technology to Support Expectant and Parenting Youth through Case Management: Lessons Learned in the Field

**DOI:** 10.1007/s10995-020-02952-0

**Published:** 2020-05-16

**Authors:** Nichole Kang, Morgan Patrick, Frances Williams, Katharine Hemady, Mara Aussendorf, Livia Greenbacker, Allison Kannam

**Affiliations:** 1 Health Promotion Council of Southeastern Pennsylvania, Inc., Philadelphia, PA 19102 USA; 2 Department of Epidemiology, Mailman School of Public Health, Columbia University, New York, NY USA; 3 Research & Evaluation Group, Public Health Management Corporation, Philadelphia, PA USA

**Keywords:** Health navigation, Case management, Information and communication technology, Young parents

## Abstract

**Introduction:**

The Support. Empower. Learn. Parenting Health Initiative (SELPHI) provides expectant and parenting youth ages 16–24 in Philadelphia with supports to improve educational, social, and economic outcomes to shape their health and the health of their children. Phone, text, video-based, and social media communication technology is built in to SELPHI’s program design to facilitate case management and connect clients to a broad referral network. Given the novelty of using information and communication technology (ICT) in case management, the reported lessons learned seek to give providers a specific and nuanced picture of ICT in case management.

**Methods:**

In its initial 6-month implementation period, SELPHI’s five case managers, called Navigators, served 59 clients. Data from feedback surveys and case records were collected from clients and Navigators. Data included client demographic characteristics, needs assessment, and contact records to inform continuous quality improvement (CQI).

**Results:**

ICT’s benefits included having multiple ways to connect to difficult-to-reach clients, the ability to be more responsive to clients, and the flexibility to address scheduling and transportation barriers. ICT’s challenges are related to Navigators’ boundary setting, limitations on rapport building, and data security considerations. CQI data are presented to illustrate the lessons learned. Text messages were the most prevalent ICT; phone calls were most successful in engaging clients. Clients’ ICT preferences differed by purpose of communication.

**Discussion:**

Findings suggest that programs should understand the nuances of client contact preferences. To maximize the benefits of ICT, programs must develop or adapt protocols based on preference and purpose of communication.

## Significance Statement

Past research on young adult populations vulnerable to health risks suggests that integrating information and communication technology (ICT) into case management may be a promising method to engage clients. The small amount of existing literature on this topic is limited to homeless youth and the use of ICT has not been studied with expectant and parenting youth. Lessons learned from the SELPHI project suggest that communication technology constitutes a useful tool to engage young adults in case management services, although some methods may be more fitting than others based on client preference and the message being conveyed.

## Introduction

Poverty, lack of educational attainment, and relationship challenges are associated with both an increased risk of unplanned pregnancy and poorer outcomes for expectant and parenting youth and their children (Kornfeld et al. 2012; Manlove 1998; Penman-Aguilar et al. 2013). In turn, teen pregnancy and parenthood can negatively impact employment and future earnings (Lee 2010) and contribute to relationship and family challenges, including children’s entry into the foster care system (Centers for Disease Control and Prevention 2015; Rodgers & McGuire 2012). Case management and health care navigation have been shown to improve academic and relationship outcomes for at-risk youth (Parise et al. 2017) in addition to physical and mental health outcomes among parenting youth (Hodgkinson et al. 2014).

Using information and communication technology (ICT), including email, text messaging, video chatting, and social media messaging with devices such as laptops and smartphones, has been recognized as a strategy to increase efficiency, collaboration, organization, and access to information for case managers (Perron et al. 2010). Electronic client records are also increasingly being integrated into social work (De Witte et al. 2016). Phone, text, video-based, and social media communication technology is highly utilized among young people today (Smith, 2011; Villanti et al. 2017); ICT is therefore a practical means to deliver services to this population.

Regular communication and retention in case management programming can be a challenge with young parents. Expectant and parenting youth are faced with many competing priorities, from child care to school schedules (Asheer et al. 2014), so sustained contact to engender retention in programming is a priority for case management programs. It can be difficult to maintain contact with clients whose phone numbers change frequently and whose cellular service plans are expended monthly. As such, program staff serving young parents have to be flexible and persistent to maintain contact with their clients; the flexibility afforded by ICT can support communication with those clients who have regular access to cellular phones and other internet-connected devices (Devine et al. 2015). Past research on a vulnerable population of homeless youth found that integrating technology had promising implications for case management (Bender et al. 2015): a large majority of youth described electronic communication with case managers positively and reported an increase in convenience, connection, accessibility, and accountability. However, existing research has yet to consider the implications of integrating ICT into case management for expectant and parenting youth.

The Support. Empower. Learn. Parenting Health Initiative (SELPHI) is a case management program implemented in Philadelphia, Pennsylvania, that employs case managers, called Navigators, to provide expectant and parenting youth (ages 16–24) with supports to improve educational, social, and economic outcomes in order to shape the health of young parents and their children. SELPHI’s case management services include linking youth to primary health care services for the entire family; social services (e.g., education and employment services, Supplemental Nutrition Assistance Program, child care assistance); parenting and healthy relationship education; and concrete supports (e.g., diapers, transportation). SELPHI provides concrete supports directly to parents when they are not available elsewhere. The intended duration of SELPHI navigation is three to 9 months, with a minimum of one in-person meeting per month. At intake, the Navigator and client determine the client’s preferred method of contact, which can change during the course of participation. The frequency of engagement is determined by clients’ needs and is established through an intake assessment that measures gaps in the services clients are receiving. Clients with lower need for support from Navigators receive ICT communication at least once per week, whereas those clients with a higher need receive ICT communication several times a week and meet in person at least every two weeks. Though these guidelines are in place, the balance of in-person versus ICT-aided case management has varied by case.

SELPHI uses various ICT to facilitate case management and connect clients to a broad referral network within the community; its goal is to maximize the number of clients that can be reached and mitigate clients’ barriers to receiving services and resources. ICT methods include phone calls, text messages, video calls (using the live-video function on cellular phones), Facebook messaging (client and Navigator send messages through Facebook’s Messenger application once the client has “followed” the SELPHI Facebook page or become Facebook Friends with their Navigator), and email (between the Navigators’ work email and the clients’ personal email). Navigators have utilized ICT to share information with clients about resources and services available to them. Text messaging and social media are popular methods for sharing detailed information, such as upcoming events and the location and contact information of referral sites.

Given the promise and novelty of using ICT in case management, the lessons learned and continuous quality improvement (CQI) data reported here may aid new programs in planning and compiling best practices. We sought to answer the following programmatic questions:

1. Which types of contacts were most common? Did participant engagement vary by contact mode?
2. What are the benefits and limitations to the use of ICT in case management with expectant and parenting youth?

The current evaluation seeks to go beyond discussing the promise of ICT to provide a specific and nuanced picture of our lessons learned around the use of ICT in case management.

## Methods

### Design

Lessons learned were derived by the program manager through supervision conversations with Navigators about client cases, challenges, and successes. This manuscript presents CQI data related to stakeholders’ experiences and opinions as relevant to the lessons learned. Data presented are from the electronic case management database and from surveys of Navigators and clients. The case management database, hereafter referred to as “the database,” contains case management records for each client, hereafter referred to as “the client record.” The design of the database was guided by both case management and evaluation needs to allow evaluation to wrap around the program. The evaluation was reviewed by the appropriate Institutional Review Board and determined not to be human subjects research.

### Participants

#### Navigators

SELPHI employed five Navigators whose caseloads ranged from 3 to 22 clients over the course of the 6-month implementation period. Navigators’ ages ranged from 24 to 54, and they had an average of 7 years’ experience in case management (1 year to 15 years, n = 4). Only four Navigators were employed at the time this information was collected.

#### Clients

Eligible individuals were between ages 16 and 24 years old and expecting a child or currently parenting at least one child. Clients’ engagement was initiated and maintained by embedding Navigators at partner organizations, including an alternative high school and two child welfare agencies, and by seeking referrals from community organizations. SELPHI served 59 clients over the course of the 6-month implementation period (January through June 2018). This period of evaluation was selected to provide early insight into program implementation; the program has since been funded for an additional 2 years. Client demographic characteristics are shown in Table 1.

**Table 1:** SELPHI client demographics (N = 59)

Characteristic	%	n	Mean	(SD)
Age			19.84	(1.61)
Number of children			1.14	(0.63)
Female	88.1	52		
Parent status				
Expectant	10.2	6		
Parenting	78.0	46		
Both	11.9	7		
Race				
African American	83.1	49		
Bi/multiracial	5.1	3		
Another race	8.5	5		
Refused	3.4	2		
Ethnicity				
Hispanic	15.3	9		
Non-Hispanic	81.4	48		
Refused	3.4	2		
Sexual orientation				
Heterosexual	89.8	53		
Gay or lesbian	3.4	2		
Bisexual	5.1	3		
Refused	1.7	1		
Highest education level				
Less than high school	10.2	6		
Currently in high school	25.4	15		
High school diploma or GED^a^	47.5	28		
Some college	16.9	10		

^a^General education diploma

### Data Sources

#### Client Records

All client records constitute data collected by client or Navigator report, including client demographic characteristics, needs assessment, and frequency and mode of contact. Navigators collected demographic characteristics and needs assessment from clients via a semi-structured interview at intake; these data were coded categorically, and frequencies were calculated to describe the client population and identify its most prevalent needs. Within client records, Navigators kept a contact log for each contact attempt made with a client. Data collected in this log included the date of contact; communication method (call, text, video call, email, Facebook message, in person, or other); outcome of contact attempt (i.e., engaged client or not); and reason for contact. Frequency of contact was aggregated by communication method and client.

#### Navigator Feedback

Four SELPHI Navigators were surveyed in the winter of 2018 (with the 19-item Navigator Feedback Survey) and summer of 2018 (13 items) to glean the perceived benefits and role of ICT in case management with this population. Survey questions solicited feedback about Navigators’ preferred mode of communication to initiate relationships, ICT’s usefulness in maintaining relationships with clients, the overall usefulness of ICT for overcoming barriers such as transportation and time commitments, and successes and challenges related to using ICT in SELPHI. Additionally, in July 2018, the four Navigators completed a 36-item open- and closed-ended survey to provide feedback about the evaluation measures included in the client database and data collection procedures (i.e., perceptions of survey burden on clients, clarity of questions, and utility of scales); perceptions of their work and relationships with clients; goals established and client progress toward achieving those goals; successes and barriers for clients and Navigators; and services they had provided. Only four Navigators were employed at the time of the winter survey: one was hired between surveys, and one had resigned by the summer survey. These surveys were all completed online. Results are informed by the feedback gathered from Navigators on the utility of ICT for better supporting their clients.

#### Client Feedback

Clients (n = 15) completed the 15-item SELPHI Client Satisfaction Survey in June 2018 to provide their perspectives on ICT and experiences with SELPHI’s services. Clients who responded to the survey did so anonymously and responded to demographic questions on age (mean = 20.50, SD = 2.14) and gender (93.3% female). Clients rated the degree to which SELPHI helped them in various domains, rated satisfaction with their Navigator’s services and use of ICT, provided feedback on their preferred ICT mode for specific purposes, and gave open-ended feedback on specific aspects of SELPHI. Clients were asked to complete the survey via an online link that Navigators texted or emailed to them, or they completed a paper survey. The uncertainty of future programming at the time the survey was collected likely contributed to the low response rate; clients became aware of this uncertainty when Navigators conducted discharge activities for each client. Discharge activities included attempting to connect active clients with other services in order to provide continuity of care should services be discontinued. Descriptive statistics on items related to client preferences for modes of contact based on contact purpose inform our results.

## Results

SELPHI used ICT to support program implementation. Navigators contacted clients via phone, text, email, video call, and social media messaging and recorded their contact attempts in the database. SELPHI enrolled 59 clients; Navigators made a total of 799 contact attempts using ICT and an additional 150 in-person contacts. Most SELPHI clients engaged in at least one in-person meeting with their Navigator, with the average client meeting face to face between three and four times.

Within the 6-month implementation period, stakeholders learned several lessons about the role that technology can and cannot play in case management with expectant and parenting youth and found both successes and challenges with using ICT for these services.

### Benefits and Successes

#### Flexibility

The use of ICT gave Navigators more flexibility; all Navigators reported that ICT either slightly or significantly reduced transportation and time barriers for clients. Additionally, Navigators stated that these modes of contact could be leveraged to provide more effective services. For instance, one Navigator stated that ICT allowed her to connect with clients on a more regular basis than if she relied on face-to-face communication or just one mode of technology. Additionally, client feedback indicated a preference for different types of contact modes depending on the type of information that was being shared. The SELPHI Client Satisfaction Survey showed that clients most prefer to make appointments via text messaging (100.0%), with fewer preferring making appointments via phone calls (80.0%) and fewer yet via email (46.7%). However, when asked how they would prefer their Navigator to check in with them about goal progress, clients reported an equal preference for texts and phone calls (86.7%) among ICT forms.

The contact log provided frequency and engagement data for each technology (Table 2). Engagement was defined as a client providing a response to a Navigator’s contact attempt (e.g., reply to a text message, answer a phone call) and engaging in a dialogue that allowed the Navigator to provide case management as a result of that contact. Text messaging was most widely used to contact clients but had the third-highest engagement rate behind phone calls and video calls. This finding, however, may simply reflect the fact that a client can receive information via text message without engaging, whereas phone calls and video calls must be answered for information to be transmitted.

**Table 2:** Contact success by mode of information and communication technology (ICT)

Mode of ICT	Number of contact attempts	Number of contact successes	Success rate (%)
Phone	95	78	82.1
Video call	8	6	75.0
Text	656	434	66.2
Facebook message	6	3	50.0
Email	34	10	29.4
Total	799	531	66.5

A total of 150 in-person contacts were recorded; because of the nature of in-person meetings, all are considered “successful”

#### Multiple Modes of Access

Having multiple contact methods for each client avoids gaps in case management services. In the Navigator Feedback Survey, three of the four Navigators identified lapsed phone service as a communication barrier for their clients and reported that this barrier was mitigated by access to multiple communication methods, such as email or Facebook. Disconnected phones are a common occurrence for the target population; the Navigators’ previous experience working with youth prepared them for this challenge and taught them to thoroughly document client contact information, attempt all the available modes of contact, and record all contact attempts.

### Challenges and Limitations

#### Limited Early Rapport Building

Making initial contact through ICT was necessary in some cases, but doing so can cause difficulty in establishing rapport with a client. Although three Navigators shared success stories demonstrating ICT as a useful method for maintaining relationships between in-person meetings, they stressed that technology cannot replace in-person interaction for building rapport with a new client. One Navigator reported, “Sometimes there is a disconnect with text—however, that just allows you to figure out what communication styles work best for clients.”

#### Potential for Breaching Boundaries

Using ICT typically allows for around-the-clock communication, which may be burdensome to staff, especially during non-work hours. One Navigator reported “almost always” feeling pressured to respond to clients immediately when they reach out, even on her own time. However, the three other Navigators’ responses ranged from “almost never” to “sometimes” feeling pressured to respond immediately. This mix of responses shows that Navigators can establish boundaries, though the potential for burden exists. Supervisors should be aware of this potential and support their staff accordingly.

#### Data Security in Communication Technology Platforms

The use of ICT has the potential for unsecured sharing of protected health information because many of the commonly used forms of ICT are not Health Insurance Portability and Accountability Act (HIPAA) compliant. SELPHI leadership made a programmatic decision to use common ICT platforms rather than separate HIPAA-compliant platforms that would have been unfamiliar to clients. All respondents to the Navigator Feedback Survey reported that clients “often” or “almost always” disclosed personal information with them; therefore, it became necessary for SELPHI to create guidelines for dealing with and avoiding the transfer of protected health information over non-secure platforms. Clients were asked to complete a consent form that made clear the potential risks of communicating via technology. Navigators used company-issued phones and computers, which included passcode-protected lock screens and software that would wipe data from the phone/computer if lost or stolen, and disabled text preview options. Navigators were directed to eliminate the use of personal identifiers including names, addresses, telephone numbers, email addresses, and Social Security numbers when communicating with clients; this information was stored separately in the database, which is HIPAA compliant.

## Discussion

Both the benefits and challenges of using ICT for case management should be considered when designing programs. The purpose of this paper is to share lessons learned within a specific program, population, and context. Program designers can maximize the benefits of incorporating ICT by scanning the experiences of other programs. Only when these limitations are addressed or minimized can ICT meet the promise of increased communication and connection with clients touted in previous literature.

Effective communication is a key driver of the success of public health programming. It can be difficult to regularly communicate with clients in case management programs; cell phone data can be turned off by the provider once the usage limit is reached, client and staff schedules may not align, and clients are often balancing a lot of priorities that come before communicating with their case manager.

ICT allowed SELPHI Navigators to communicate with clients using technology that both parties already use in their everyday lives and allowed for myriad contact methods in case a client did not have reliable phone access. Over the course of the first year of programming, staff learned several lessons about the capabilities and challenges of using ICT in a youth-targeting case management program. Programs should aim to understand the preferred modes of communication of their target populations and develop or adapt communication protocols based on those preferences. Staff serving youth should be responsive to individual clients’ preferred modes of communication. Additionally, staff should consider which mode of contact is likely to result in engagement and to build rapport when such outcomes are critical to impactful case management. For instance, clients generally reported a preference for text messaging over other modes for making appointments, but they preferred texting equally to phone calls for discussing their progress toward goals. This may be preliminary evidence that different modes of ICT can best address different contact purposes and that clients’ preferences differ.

The lessons learned from this program were generated from the first 6-month implementation period of a single program; as such, they are not designed to be generalizable and should be considered alongside lessons learned from other programs when designing programs. Many of the findings from the CQI data contributed to the lessons learned but do not stand alone as evaluation findings. In particular, the client feedback was gathered from about one-quarter of SELPHI clients, so these results may not be representative of all SELPHI clients.

Research on ICT use in case management with expectant and parenting youth is warranted to find a balance between mode efficiency, satisfaction, and success. For example, does following an individual client’s contact method preferences result in increased engagement or satisfaction? Additionally, future research should explore whether *reduced nonverbal cues* in some ICT, such as text messaging and phone communication, may present a challenge to rapport building because of the potential for misinterpretation of tone, mood, or meaning between the individuals who are communicating. Future research may also explore whether ICT results in a reduction in the disclosure of personal information necessary to case management because of low rapport or concerns over privacy of text messages or emails.
